# Health Tract No. 325

**Published:** 1868-06

**Authors:** 


HEALTH TRACT No. 325.

### MAD DOG BITES,

Two months ago, a lady was bitten by a dog, in East Newark; the next day, the husband, in whipping the animal, was bitten on the hand; the dog was killed, the wound healed, and the circumstances were almost forgotten; but a few days since the husband experienced some difficulty in swallowing, soon fell into horrible convulsions and died. The wife is well. As all are exposed to the bites of domestic animals, cat or dog, the mind ought to be informed as to what ought to be done on the spot.

The moment any one has been bitten or stung, a rag should be thoroughly wetted with spirits of hartshorn and patted on the bitten place for an hour, then bound on, to be repeated for twenty-four hours, for two reasons; the hartshorn creates a smarting, and redness and inflammation, which keeps the blood on the surface, tends, as it were, to keep the poison on the outside, so that it may be washed out. Second, the virus of poisonous bites and stings, is an acid; hartshorn is the strongest alkali, and will antagonize an acid in an instant. If no hartshorn is at hand, take the ley of wood ashes, or even make a poultice of fresh wood ashes and water, which make an alkili, let it remain on the wound until some hartshorn can be procured.

A dog ought not to be killed if he has bitten a person, although he may appear to be mad, because such animals, when allowed to become quiet and composed, have often returned to a perfectly natural condition, and thus the mind of the person bitten has been saved from most terrible forbodings.

Medical observation shows that about one person in twenty, bitten by mad dogs, become hydrophobic. In one noted case, a dog bit twenty-one animals and persons, and but one of these became hydrophobic. Not all persons exposed even to small-pox, take it; or to cholera, measles, or any other communicable disease; showing that even dreadful diseases invade only those systems, or states of constitution, which are succeptible to their influence. A London brewer's drayman, who has been swilling several quarts, if not gallons of beer daily, for forty years, if scratched on the hand with a pin, recovers from it, if at all, very slowly, often never; sometimes death follows in a few days from convulsions or mortification. I knew a gentleman of wealth, whose foot slipping as he was stepping into his carriage, the shin-bone was thrown against the scraper or step, and he died of mortification of the limb in a few days; because he was a steady hard-drinker of whiskey, and his system had no power of re-cuperation or resistance against disease. This principle is proven by the case in hand; the husband died; the wife was unaffected. It is not impossible that if a perfectly healthy man of strong mind were bitten by a mad dog, he would not become hydrophobic; but the precautions named ought to be taken by all, even if the dog or cat be not mad.

